# Supplementary material for: Ambient Air Pollution and Acute Ischemic Stroke—Effect Modification by Atrial Fibrillation
Source: J Clin Med. 2022 Sep 15;11(18):5429. doi: 10.3390/jcm11185429 (PMC9503161; doi:10.3390/jcm11185429)
Supplement: Supplementary file 1 [file jcm-11-05429-s001.zip › jcm-1885183-supplementary.pdf]

Journal of
Clinical Medicine

MDPI

Supplementary Materials

Table S1. Modification effect of age group and smoking status on air pollutant and AIS.

Pollutant	Adjusted OR *	95%CI	*p* Value
Among patients aged ≥65 years relative to those aged <65 years			
$PM_{2.5}$	1.000	0.998–1.001	0.519
PM_{10}	1.000	0.999–1.001	0.975
O_3	1.002	1.000–1.004	0.028
NO_2	0.997	0.995–1.000	0.066
SO_2	0.996	0.993–0.999	0.008
CO	0.979	0.878–1.093	0.709
Among patients who smoked relative to those who did not smoke			
$PM_{2.5}$	0.999	0.997–1.000	0.096
PM_{10}	0.999	0.997–1.000	0.019
O_3	0.998	0.996–1.000	0.057
NO_2	1.002	0.999–1.005	0.132
SO_2	1.005	1.002–1.008	0.001
CO	0.874	0.780–0.979	0.020

* Adjusted for daily mean temperature, daily relative humidity and daily total rainfall. Abbreviations: AF—atrial fibrillation; AIS—acute ischemic stroke; CI—confidence interval; CO—carbon monoxide; NO_2—nitrogen dioxide; O_3—ozone; OR—odds ratio; $PM_{2.5}$—particulate matter 2.5; PM_{10}—particulate matter 10; SO_2—sulfur dioxide.

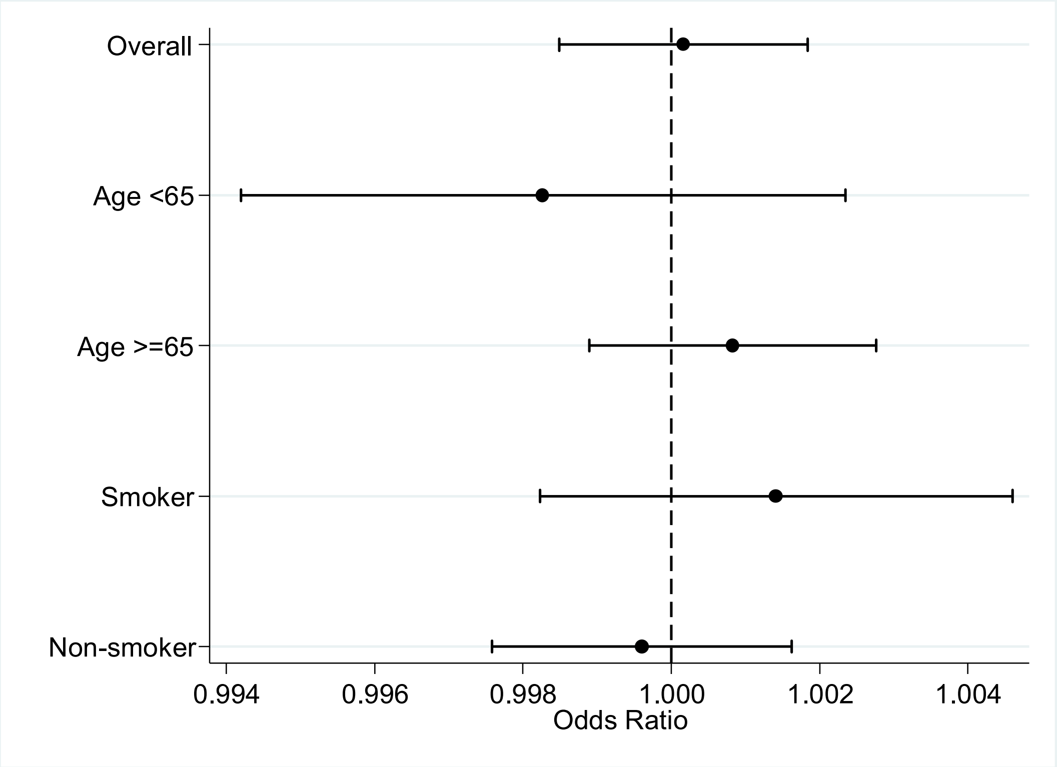

Figure S1. Adjusted odds of AF for $PM_{2.5}$ exposure among all and subgroups of AIS patients.

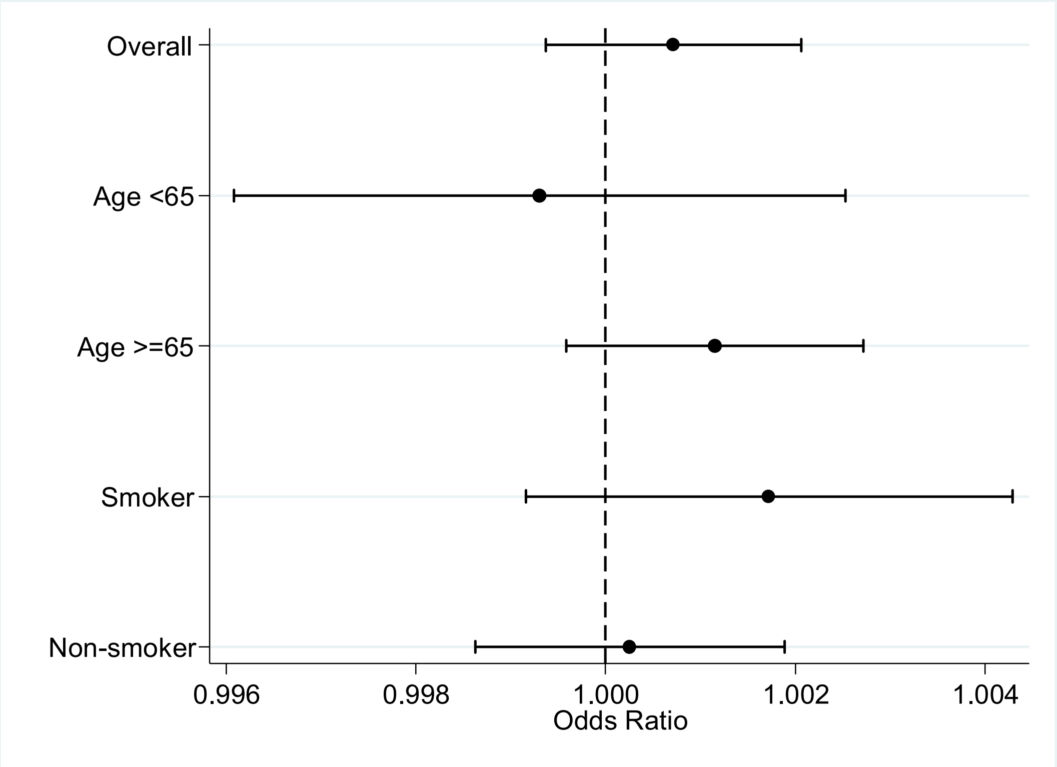

Figure S2. Adjusted odds of AF for PM_{10} exposure among all and subgroups of AIS patients.

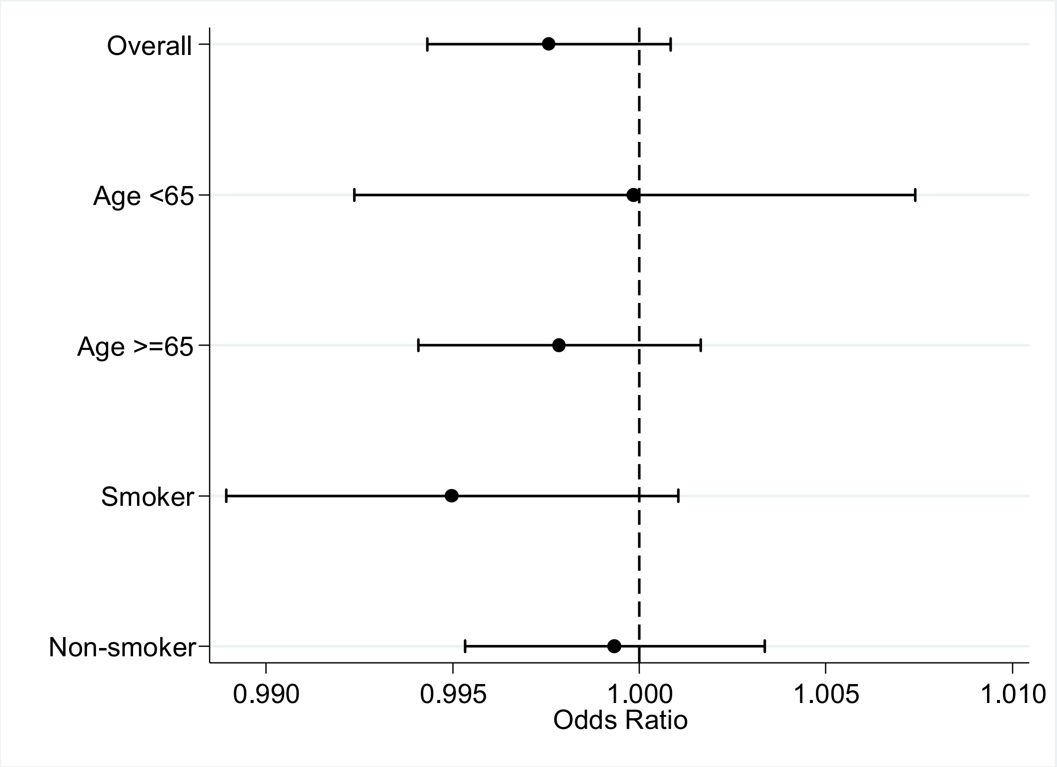

Figure S3. Adjusted odds of AF for NO_2 exposure among all and subgroups of AIS patients.
